# Biological implications of coeruleospinal inhibition of nociceptive processing in the spinal cord

**DOI:** 10.3389/fnint.2012.00087

**Published:** 2012-09-28

**Authors:** Masayoshi Tsuruoka, Junichiro Tamaki, Masako Maeda, Bunsho Hayashi, Tomio Inoue

**Affiliations:** Department of Physiology, Showa University School of DentistryTokyo, Japan

**Keywords:** locus coeruleus/subcoeruleus, coeruleospinal pathway, pain control, peripheral inflammation, startle response, air-puff stimulation, spinal dorsal horn

## Abstract

The coeruleospinal inhibitory pathway (CSIP), the descending pathway from the nucleus locus coeruleus (LC) and the nucleus subcoeruleus (SC), is one of the centrifugal pain control systems. This review answers two questions regarding the role coeruleospinal inhibition plays in the mammalian brain. First is related to an abnormal pain state, such as inflammation. Peripheral inflammation activated the CSIP, and activation of this pathway resulted in a decrease in the extent of the development of inflammatory hyperalgesia. During inflammation, the responses of the dorsal horn neurons to graded heat stimuli in the LC/SC-lesioned rats did not produce a further increase with the increase of stimulus intensity in the higher range temperatures. These results suggest that the function of CSIP is to maintain the accuracy of intensity coding in the dorsal horn because the plateauing of the heat-evoked response in the LC/SC-lesioned rats during inflammation is due to a response saturation that results from the lack of coeruleospinal inhibition. The second concerns attention and vigilance. During freezing behavior induced by air-puff stimulation, nociceptive signals were inhibited by the CSIP. The result implies that the CSIP suppresses pain system to extract other sensory information that is essential for circumstantial judgment.

## Introduction

It is a general principle that the brain regulates its sensory inputs. This principle applies to all of the somatosensory pathways that have been investigated. The inhibitory regulation of nociceptive inputs is of particular clinical interest because this regulation may lead to a reduction of pain. Inhibitory action on nociceptive processing is accomplished via descending or ascending inhibitory pathways (Horie et al., 1991; Koyama et al., 1995; Willis and Coggeshall, 2004). There is considerable interest in the role of descending inhibitory pathways and the possibility of targeting these pathways for clinical treatments. A number of studies have demonstrated that stimulation at many sites of the brain can produce analgesia by inhibiting nociceptive transmission in the spinal cord (see review by Willis and Coggeshall, 2004).

The descending pathway from the nucleus locus coeruleus (LC) and the nucleus subcoeruleus (SC) is one of centrifugal pain control systems. The LC/SC provides noradrenergic innervation of the spinal cord (Guyenet, 1980; Westlund et al., 1983, 1984; Fritschy and Grzanna, 1990; Clark and Proudfit, 1991, 1992; Grzanna and Fritschy, 1991; Proudfit and Clark, 1991). Activation of the LC/SC either electrically or chemically can produce profound antinociception (Segal and Sandberg, 1977; Margalit and Segal, 1979; Jones and Gebhart, 1986a; Jones, 1991; West et al., 1993) and can inhibit nociceptive activity in dorsal horn neurons (Hodge et al., 1981; Mokha et al., 1985; Jones and Gebhart, 1986a,b, 1987, 1988). Thus, the coeruleospinal inhibitory pathway (CSIP) appears to play a significant role in spinal nociceptive processing.

During the first decade of the twenty-first century, we were particularly interested in the role of the CSIP in the everyday life of mammals, including its roles in normal and abnormal pain condition. Based on our experimental results that characterized coeruleospinal inhibition of nociceptive processing in the spinal cord, this review provides an answer to the question regarding the role of coeruleospinal inhibition in the mammalian brain. We hope that our inferences will aid in a better understanding of the role of centrifugal control of sensation.

## Contribution of the CSIP to pain control under an abnormal pain state and its biological implications

### Activation of the CSIP by peripheral inflammation (Tsuruoka and Willis, 1996 a,b)

In a series of our study, inflammatory pain, but not neuropathic pain, was used as an abnormal pain state. Pain can divide three groups (i.e., inflammatory pain, neuropathic pain, and psychogenic pain) on the basis of the source, nociceptive, neuropathic, and psychogenic pain. Inflammatory pain is nociceptive pain via nociceptor, neuropathic pain is a morbid pain induced by dysfunction of the peripheral or central nervous system, and psychogenic pain results from the psychological reasons. We adopted inflammatory pain because peripheral inflammation is a matter of frequent occurrence in compared to other pain in everyday life.

**Figure 1:**
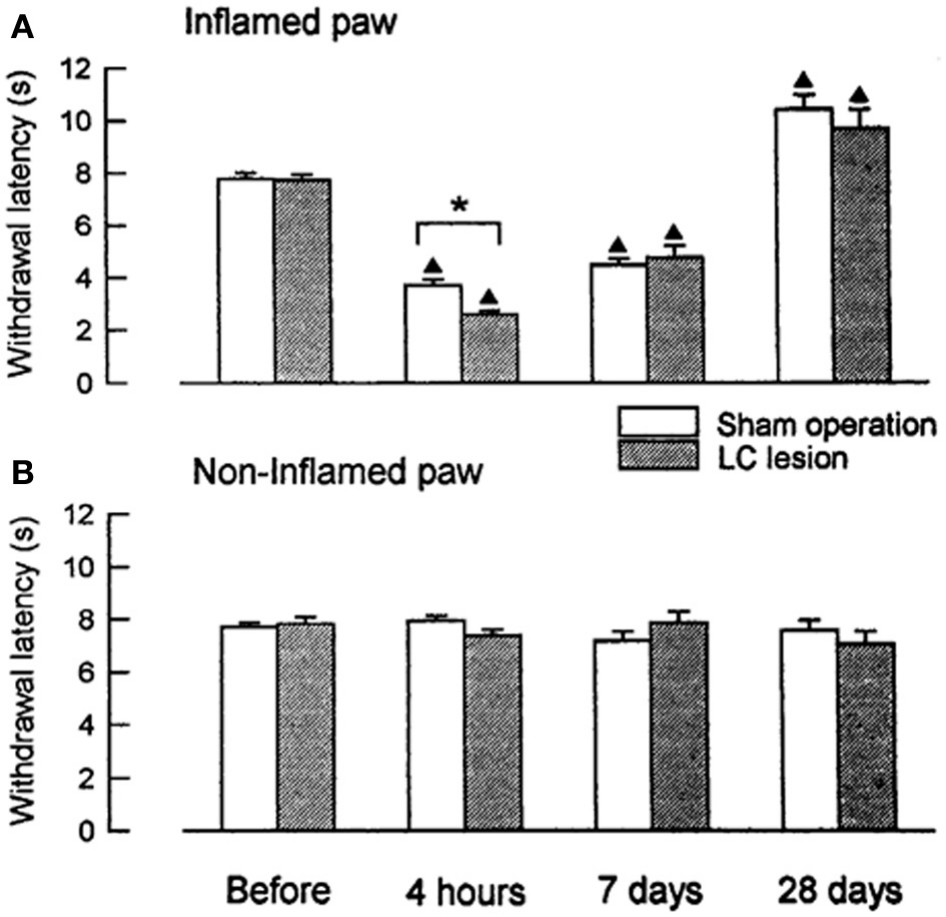
**Changes in PWLs following unilateral injection of carrageenan in LC-lesioned (*n* = 9) and sham-operated rats (*n* = 20). (A)** The inflamed paws. **(B)** The contralateral non-inflamed paws.^▲^*P* < 0.01, significantly different from PWLs before injection. ^*^P < 0.01, significantly different between two groups of rats (Tsuruoka and Willis, 1996b).

We compared the development of peripheral hyperalgesia between rats that received bilateral lesions to the LC/SC and sham-operated, control animals for 4 weeks after administration of carrageenan (an inflammatory agent) (Figure 1). Four hours after the induction of inflammation, the paw withdrawal latencies (PWLs) to heat stimuli in the inflamed paws of the LC-lesioned rats were significantly shorter than those of the sham-operated rats. This result shows that peripheral inflammation activates the CSIP and that the activation of this pathway results in a decrease in the extent of the development of hyperalgesia. The difference in the PWLs between the two groups was not observed at 7 days, whereas, edema and hyperalgesia were still present in the inflamed paw. This result suggests that the CSIP is active only in the acute phase of the inflammatory process.

### A possible interaction with opioid systems (Tsuruoka and Willis, 1996b)

**Figure 2:**
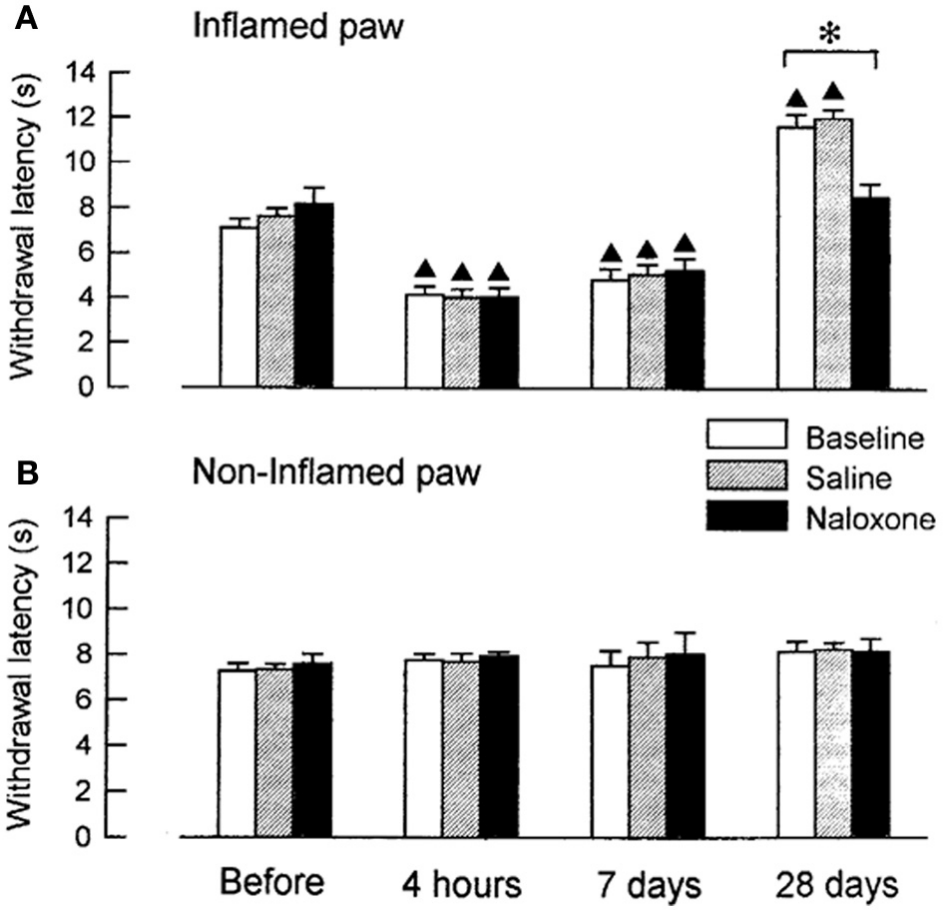
**The effect of naloxone or saline on PWLs in sham-operated rats tested 4 h, 7 days and 28 days after the injection of carrageenan.** The data were obtained 10 min after intraperitoneal (i.p.) naloxone (*n* = 8) or saline (*n* = 8) and are presented for both the inflamed **(A)** and the contralateral non-inflamed **(B)** paws.^▲^*P* < 0.01, significantly different from PWLs before injection. ^*^P < 0.01, significantly different between two groups of rats (Tsuruoka and Willis, 1996b).

**Figure 3:**
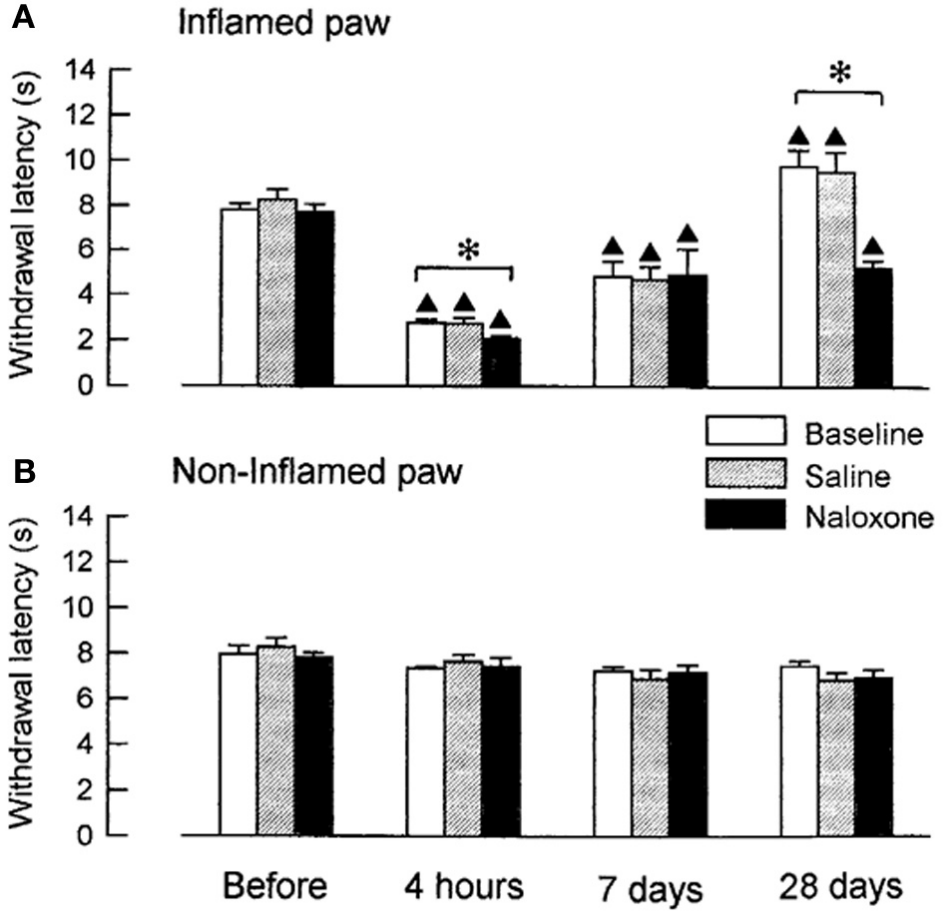
**The effect of naloxone or saline on PWLs in LC-lesioned rats tested 4 h, 7 days and 28 days after the injection of carrageenan.** The data were obtained 10 min after i.p. naloxone (*n* = 6) or saline (*n* = 6) and are presented for both the inflamed **(A)** and the contralateral non-inflamed **(B)** paws.^▲^*P* < 0.01, significantly different from PWLs before injection. ^*^P < 0.01, significantly different between two groups of rats (Tsuruoka and Willis, 1996b).

We examined, whether coeruleospinal inhibition of nociceptive processing depends on an interaction with other inhibitory systems that involve opioid peptides (Figures 2, 3). In the acute phase of inflammation, systemic administration of naloxone significantly further decreased the PWLs of the LC-lesioned rats, which indicate that opioid inhibitory mechanisms are active in the acute phase of inflammation. This result suggests that the coeruleospinal inhibition system interacts with the opioid inhibitory system. However, systemic naloxone never reversed nociceptive threshold in sham-operated rats under inflammation, whereas reverse effects observed in LC-lesioned rats. These results indicate that the coeruleospinal inhibitory system is far predominant in compared to the opioid inhibitory system under inflammatory pain state.

Opioid inhibitory mechanisms were inactive in both the LC-lesioned rats and the sham-operated rats at 7 days, whereas edema and hyperalgesia were still present in the inflamed paws. Comparable data have been obtained in rats with unilateral inflammation in which naloxone induced no significant effect at 1 week, whereas this drug reduced the paw-pressure threshold 24 h after the induction of inflammation (Millan et al., 1988). In both the sham-operated and the LC-lesioned rats, we found that the baselines PWLs of the inflamed paws were prolonged for the rats that had recovered from the inflammation. These analgesic states at 28 days resulted from the activation of endogenous opioid controls, which was apparent following systemic administration of naloxone. This finding is consistent with reports on rats with carrageenan-induced inflammation (Kayser and Guilbaud, 1991) and rats with neuropathic hyperalgesia (Attal et al., 1990), whereby naloxone produced a hyperalgesic effect in rats that had recovered from either inflammation or neuropathic hyperalgesia.

### The role of the CSIP in the intensity coding of nociceptive signals under an abnormal pain state (Tsuruoka et al., 2003)

**Figure 4:**
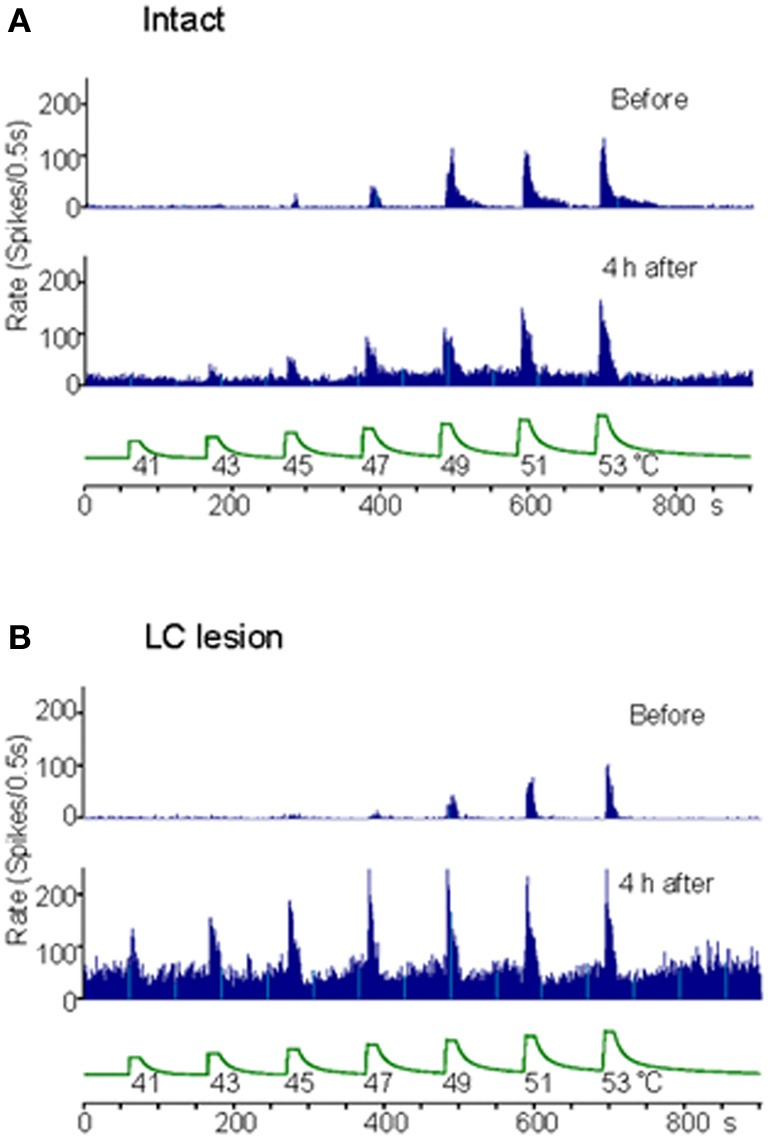
**Rate histograms for the responses to graded heat stimuli from a neuron located in the dorsal horn ipsilateral to the site of inflammation.** The responses to graded heat stimuli of a neuron were tested before and 4 h after the induction of inflammation. **(A)** Rate histograms for a neuron in a LC/SC-intact rat. **(B)** Rate histograms for a neuron in a LC/SC-lesioned rat (Tsuruoka et al., 2003).

**Figure 5:**
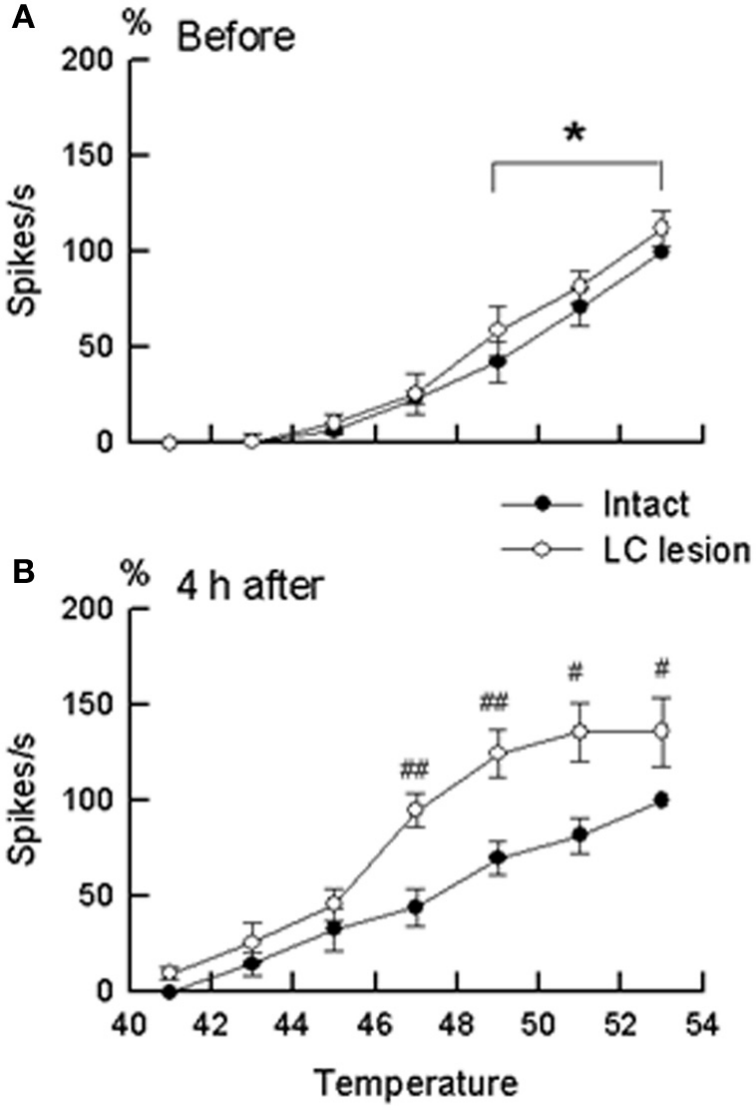
**The stimulus–response relationship in wide-dynamic-range neurons located in the dorsal horn ipsilateral to the site of inflammation. (A)** Stimulus–response relationship before the induction of inflammation. **(B)** Stimulus–response relationship at 4 h after the induction of inflammation. Closed circles (°) represent neurons in the LC/SC-intact rats (*n* = 11). Open circles (•) represent neurons in the LC/SC-lesioned rats (*n* = 20). Neuronal discharges to each temperature in graded heat stimuli (ordinate) are expressed as a percentage of the control. In **(A)** and **(B)**, 100% (control) were discharges to heating at 53°C in the LC/SC-intact rats. ^#^*P* < 0.05, ^##^*P* < 0.01, significantly different from the value of the LC/SC-intact rats (ANOVA, with Scheffe's t-test as a post hoc analysis of differences). ^*^P < 0.05, significantly different between responses to heating at 49°C and responses to heating at 53°C (ANOVA, with Scheffe's t-test as a post hoc analysis of differences). (Tsuruoka et al., 2003).

Extracellular recordings were made from the sites at lumbar enlargement of the spinal cord that had receptive fields on the hindpaws or toes. The neurons included 63 wide-dynamic-range neurons and two high threshold neurons. These neurons were tested for changes in heat-evoked response during hindpaw inflammation (Figures 4, 5). During inflammation, the responses of the dorsal horn neurons to graded heat stimuli in the LC/SC-lesioned rats did not produce a further increase with the increase of stimulus intensity in the higher range temperatures (49–53°C), whereas the responses recorded from the LC/SC-intact rats continued to increase at temperature of 49°C or higher. Therefore, it is clear that the plateauing of the heat-evoked response in the LC/SC-lesioned rats during inflammation is due to a response saturation that results from the lack of coeruleospinal inhibition.

Previous studies have reported that the descending system from the brain stem, including the LC/SC, becomes more active in modulating spinal nociceptive processes during peripheral inflammation (Ren and Dubner, 1996; Wei et al., 1999). In these studies, it has been suggested that the CSIP plays a role in suppressing the hyperexcitability of nociceptive dorsal horn neurons during inflammation. Our study provides additional findings concerning the role of coeruleospinal inhibition in nociception under the condition of inflammation. In intensity coding, the plateauing of the stimulus–response curve of dorsal horn neurons indicates that the accuracy of the transmission of stimulus intensity decreases in the dorsal horn. This implies that the difference of stimulus intensity in higher temperature ranges cannot be distinguished in the LC/SC-lesioned rats in which the plateauing of the heat-evoked response was observed. Because the plateauing of the heat-evoked response was not seen in the LC/SC-intact rats, the CSIP activated by peripheral inflammation may be involved in the prevention of the plateauing of the heat-evoked response in the dorsal horn. Activation of the CSIP induces a decrease of activity in response of dorsal horn neurons to noxious heating so that the heat-evoked responses do not produce response saturation in the range of higher temperatures which can prevent the plateauing of the heat-evoked response in the dorsal horn. It seems that the function of CSIP activation by peripheral inflammation is to maintain the accuracy of intensity coding in the dorsal horn. Thus, a possible role of CSIP activation by peripheral inflammation is to provide a means to discriminate among differences in the intensity of a painful stimulus in an inflamed region, as well as in the condition without inflammation. It is likely that the CSIP contributes to the discrimination of the intensity of pain sensation under abnormal pain states, such as inflammation.

### Coeruleospinal inhibition on visceral pain processing and visceromotor reflexes (Tsuruoka et al., 2010b)

**Figure 6:**
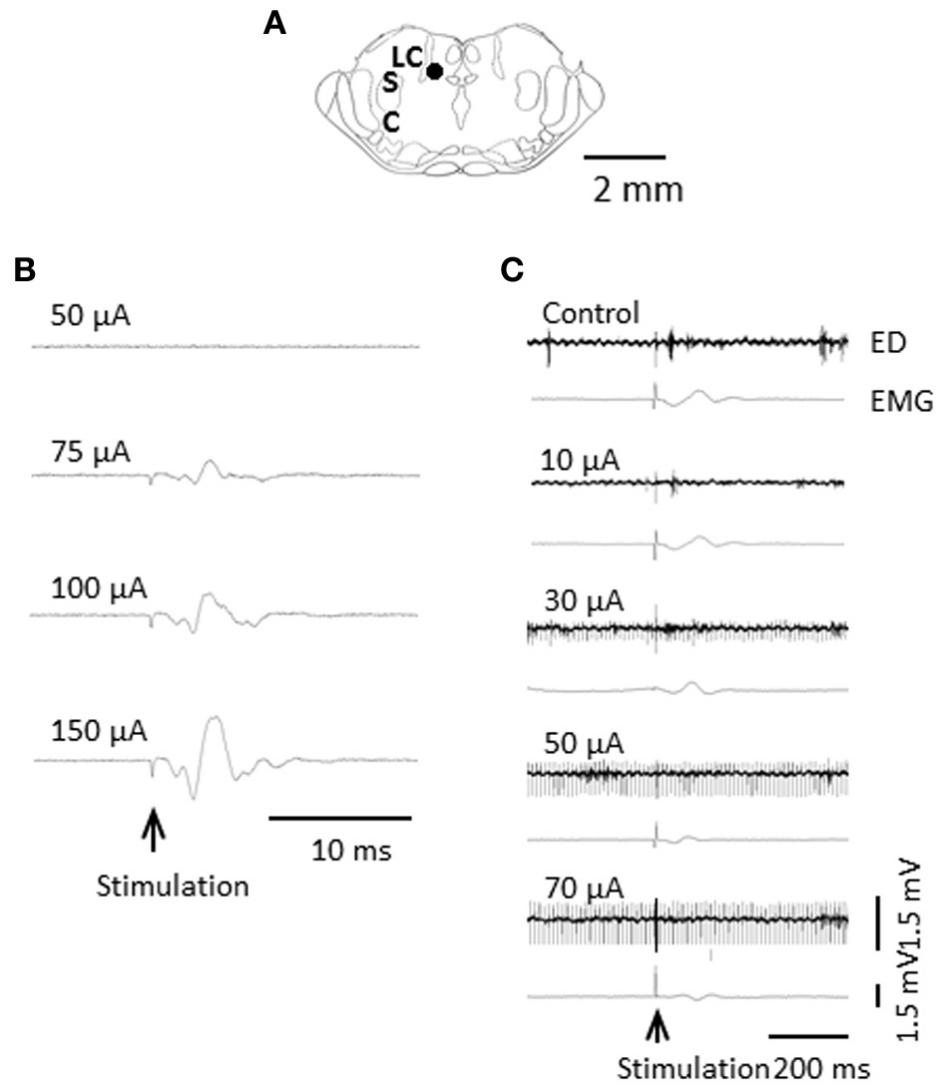
**Difference in inhibitory effect of LC/SC stimulation between the evoked discharge and the EMG activity. (A)** Stimulation site of the LC/SC (closed circle). **(B)** The EMG activity in the masseter muscle evoked by an increase in the intensity in LC/SC stimulation. **(C)** An example of the effect of graded LC/SC stimulation on the evoked discharge and the EMG activity. Note that LC/SC stimulation at a stimulus intensity below 50 μA never produced EMG activity of the masseter muscle associated with stimulation of the mesencephalic trigeminal nucleus, located just lateral to the LC/SC, and that EMG activity was still observed even when the evoked discharge was completely inhibited by LC/SC stimulation at an intensity over 50 μA. Electrical stimulation of the descending colon is indicated by the arrow. (Tsuruoka et al., 2010b).

**Figure 7:**
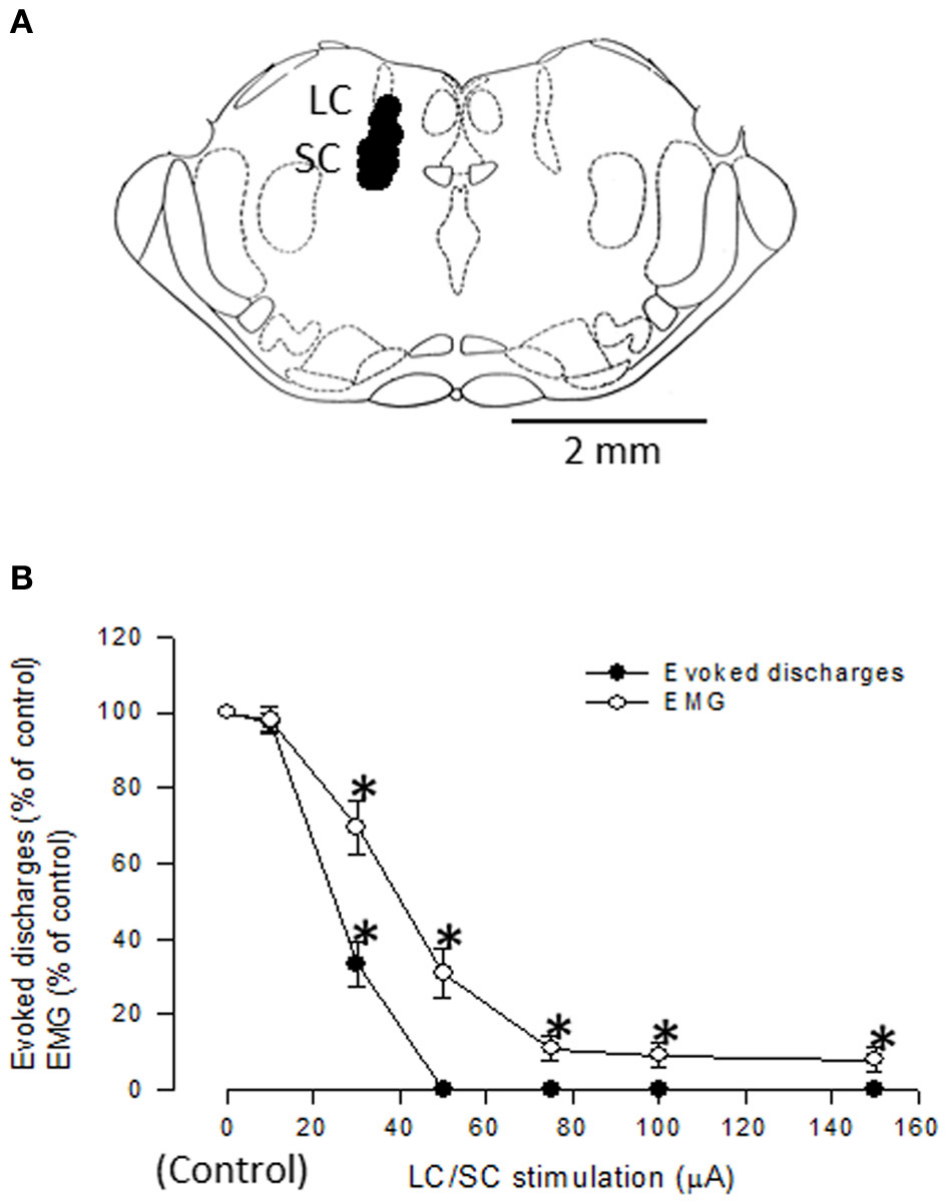
**(A)** Localization of LC/SC stimulation sites (*n* = 12). Each closed circle represents one animal. The rostrocaudal extension of the stimulation sites was 9.6 ± 0.1 mm caudal to the bregma. Only data from rats in which the tip of the stimulating electrode was located within the LC/SC were adopted. **(B)** Graphs summarizing the effects of varying stimulus intensities of LC/SC stimulation on either the evoked discharge or the EMG activity (*n* = 8). The evoked discharge and the EMG activity during LC/SC stimulation are expressed as a percentage of the value before LC/SC stimulation (control). Note that the inhibitory effect was different between the evoked discharge and the EMG. ^*^P < 0.05, significantly different from the control. (Tsuruoka et al., 2010b).

Visceral nociceptive signals are the subject of coeruleospinal inhibition (Liu et al., 2007). We identified, in rats, dorsal horn neurons whose visceral nociceptive responses were not inhibited by the CSIP (LC/SC-unaffected neurons) (Liu et al., 2008). To determine the possible role of LC/SC-unaffected neurons in pain processing and visceromotor reflexes (muscular defense), we electrically stimulated the descending colon, and simultaneously recorded both the evoked discharge in the ventral posterolateral (VPL) nucleus of the thalamus and the electromyogram (EMG) of the abdominal muscle under halothane anesthesia (Figures 6, 7). It is known that spinothalamic tract neurons that are excited by visceral nociceptive stimuli are located in the dorsal horn and that postsynaptic dorsal column neurons, which conduct visceral nociceptive signals in the dorsal column, are located near the central canal of the spinal cord (Al-Chaer et al., 1996, 1999; Ness, 2000; Palecek et al., 2002, 2003; Willis and Coggeshall, 2004). We clarified that all the LC/SC-unaffected neurons tested were located in the dorsal horn, and none were in the area near the central canal of the spinal cord (Tsuruoka et al., 2008). This result suggests that the LC/SC-unaffected neurons include spinothalamic tract cells. It has been confirmed that the spinothalamic tract neurons are involved in the development of visceromotor reflexes, such as muscular defense (Palecek and Willis, 2003). Thus, the LC/SC-unaffected neurons may be involved in visceromotor reflexes. As seen in Figures 6, 7, the inhibitory effect of LC/SC stimulation was different between in the evoked discharge of the VPL and the EMG of the abdominal musculature. The EMG was not completely inhibited even when the stimulus intensity was increased up to 150 μA, whereas the evoked discharge disappeared. If the LC/SC-unaffected neurons were involved in visceromotor reflexes, the presence of the LC/SC-unaffected neurons can explain the fact that visceromotor reflexes are not completely inhibited during activation of the coeruleospinal modulation system. The minimum visceromotor reflex responses (muscular defense) are maintained by the presence of LC/SC-unaffected neurons, which play an important role of protecting visceral organs.

Visceral nociceptive information ascending in the spinothalamic tract subserves multiple functions, acting as components of visceromotor reflexes as well as signals for producing visceral pain (Palecek and Willis, 2003). The location of the LC/SC-unaffected neurons in the spinal cord and the different inhibitory effects of LC/SC stimulation between the evoked discharge and the EMG responses lead to the conclusion that the LC/SC-unaffected neurons contribute to the maintenance of a minimum tonic contraction of the abdominal musculature even when visceral pain is completely inhibited. Considering a role of muscular defense, it is reasonable to assume that some visceral nociceptive neurons are not under the control of the CSIP to prevent the disappearance of muscular defense. Thus, the presence of the LC/SC-unaffected neurons may be advantageous for an individual in an abnormal pain state, such as inflammation.

## Contribution of the CSIP to pain control accompanied by the mammalian-startle response

### Involvement of the LC/SC in the induction of freezing behavior following the startle reaction (Tsuruoka et al., 2010a)

The startle response is an example of a simple behavior in mammals. An air puff is one of the startle-eliciting stimuli (Davis, 1984; Taylor et al., 1991; Knapp and Pohorecky, 1995; Cooke and Graziano, 2003, 2004; Sttensland et al., 2005; Lockey et al., 2009). The air-puff-induced startle response is widely used to study the function of the central nervous system (Geyer et al., 1982; Anand et al., 1999; Cooke and Graziano, 2003, 2004), including habituation (Rinaldi and Thompson, 1985), motor and cardiovascular responses (Retting et al., 1986; Woodworth and Johnson, 1988; Casto et al., 1989; Taylor et al., 1991; Zaretsky et al., 2003; de Menezes et al., 2008), and anxiety (Barros and Miczek, 1996). The air-puff startle reaction consists of a marked extension of both forelimbs and hindlimbs or a rapid flexion of the whole body that results in an overall shortening of the body (Cassela and Davis, 1986; Taylor et al., 1991; Knapp and Pohorecky, 1995). Following the startle reaction, rats react by freezing with a defense response of approximately 2–5 s in length. We designated this freezing behavior as a defensive-like, immobile posture (DIP) on the basis of Davis' study (1984).

Regression analysis showed a low correlation (*R*^2^ = 0.11) in the relation between the DIP period and the startle magnitude, which suggests that the DIP period is not influenced by the startle magnitude. This finding generates the notion that the DIP is an independent component of the startle response, although the startle reaction and the DIP are continuous behavioral responses in the air-puff startle. In this context, the DIP period might be used as another endpoint for assessing the air-puff startle.

The startle magnitude in the LC/SC-lesioned rats was significantly less than that before lesions, although the startle magnitude only slightly decreased. This result is consistent with preceding investigations in which an LC lesion decreased the effect of the startle reaction in rats (Adams and Geyer, 1981). Bilateral lesions of the LC/SC produced a significant reduction of the DIP period, as well as in the startle magnitude. These results suggest that the LC/SC is involved in the induction of both the startle reaction and the DIP, whereas the startle magnitude and the DIP period are independent endpoints for assessment. The reduction of both the startle magnitude and the DIP period in the LC/SC-lesioned rats suggests that the LC/SC exerts an excitatory influence on the air-puff startle.

LC/SC neurons have been implicated in regulation of attention and vigilance (Foote et al., 1983; Aston-Jones et al., 1991). The DIP period, therefore, seems to be an attentional state and a vigilance condition. This notion may be supported by the following finding reported by Knapp and Pohorecky (1995): an air-puff stimulus elicits ultrasonic vocalizations (e.g., 22 kHz), which are thought to reflect an aversive behavioral state, following the startle reaction in rats. Considering these findings, it can be inferred that following the startle reaction, rats likely focus attention on judging their circumstances so that unnecessary sensory information may be inhibited to extract other sensory information which is essential to the survival of an individual. It is likely that the DIP period is the time for circumstantial judgment.

### Activation of the CSIP due to air-puff stimulation (Tsuruoka et al., 2011)

**Figure 8:**
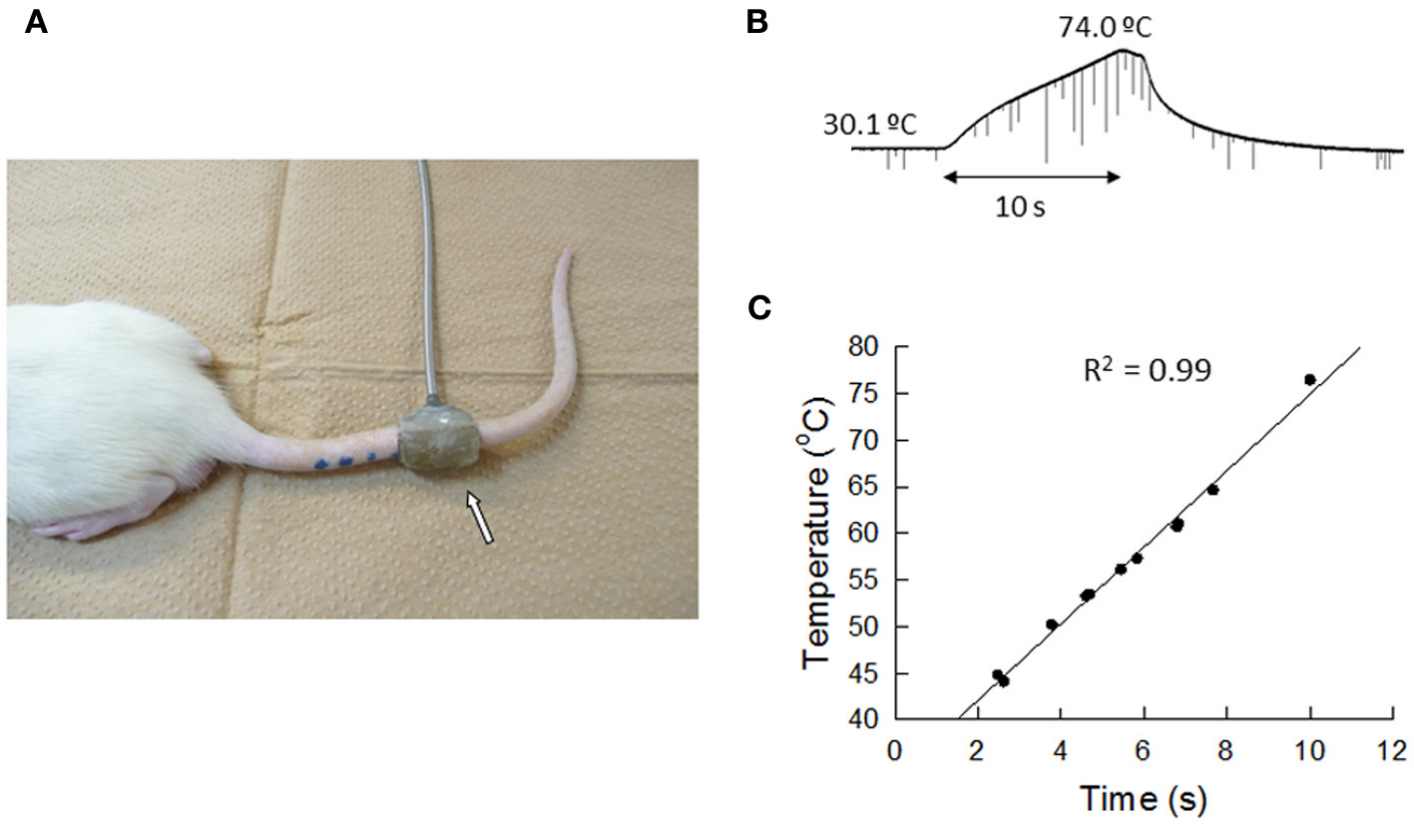
**(A)** Photograph of the tail heating. The heat source was attached to the tail, and a voltage (35 V) was applied to the coil. The heat source was a tubiform coil of wire (50 turns) covered with a thin film made of acrylic resin. The whole aspect of the heat source looked like a plastic tube with an 8-mm inside diameter and a 20-mm length. Half of the inside was the effective electric heating surface. Following heating of the tail, rats whisked their tail (tail flick reflex) and bit the heat source when the tail was unable to be removed from heating by tail flick. The arrow points to the heat source. **(B)** An example of changes in the skin temperature of the tail after the heating was begun. In this case, the skin temperature of the tail before heating was 30.1°C. After heating was begun, the skin temperature of the tail almost linearly increased to 74.0°C at 10 s. **(C)** The relation between the skin temperature of the tail and the time from the beginning of tail heating. Eleven points were obtained from 11 untreated rats. Note that a high correlation (*R*^2^ = 0.99) was observed between the skin temperature of the tail and the time from the beginning of tail heating (Tsuruoka et al., 2011).

**Figure 9:**
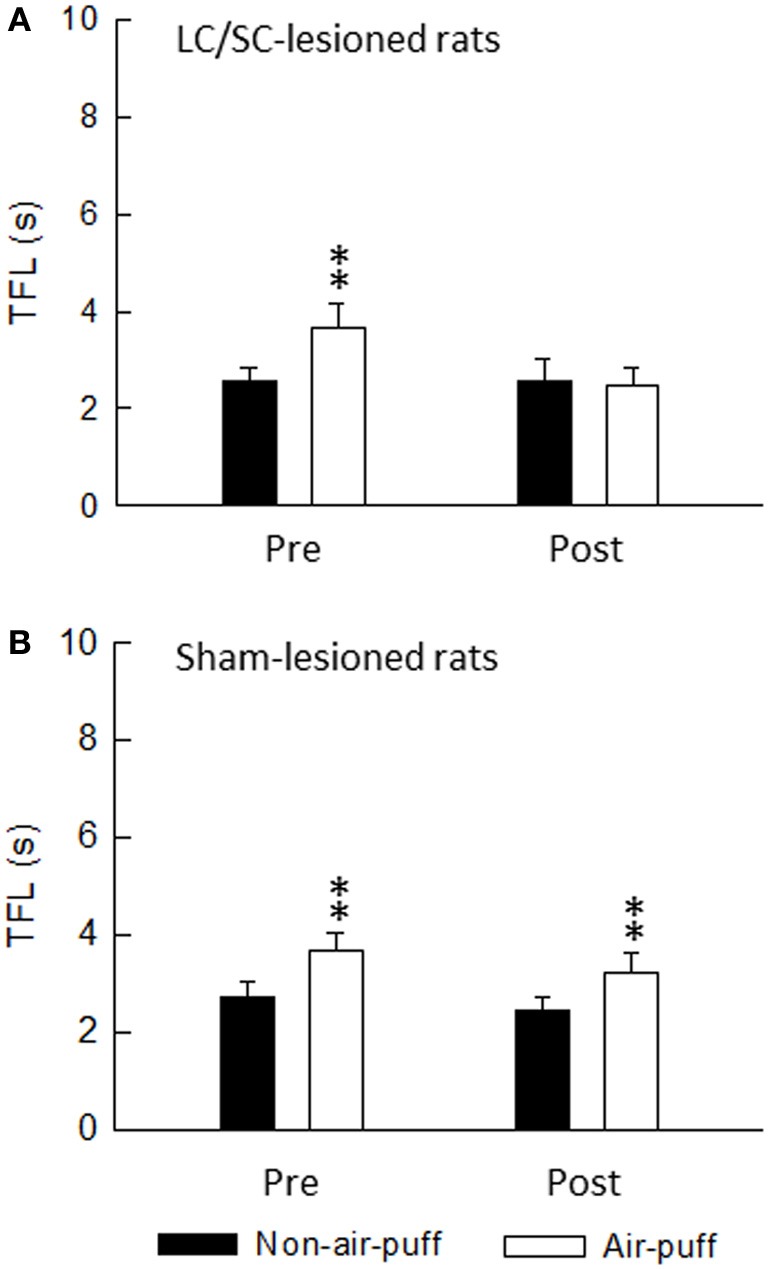
**The effect of either bilateral lesions of the LC/SC (A, *n* = 12) or sham lesions of the LC/SC (B, *n* = 10) on the tail flick latency.** ^**^P < 0.01, significantly different from the tail flick latency of the non-air-puff condition. Pre, pre-lesions; Post, post-lesions. Note that a significant Air-puff stimulation-induced prolongation of the tail flick latency was not observed in post-lesions of the LC/SC-lesioned rats, whereas tail flick latencies were significantly prolonged by air-puff stimulation in pre-lesions, suggesting that the descending inhibitory system from the LC/SC is involved in air-puff stimulation-induced antinociception (Tsuruoka et al., 2011).

Because stimulation of the CSIP produces inhibition of nociceptive transmission in the spinal dorsal horn (Tsuruoka et al., 2004; Liu et al., 2007), the tail flick test was used to examine air-puff stimulation-induced activation of the CSIP (Figure 8). The tail flick test in rats has often been used for measuring a response to a noxious stimulus and for assaying analgesic drugs, since it was first described by D'Amour and Smith (1941). The antinociceptive effect has been estimated by the prolongation of the tail flick latency (e.g., Li et al., 2010; Schröder et al., 2010; Silva et al., 2010). As shown in Figure 9, tail flick latencies with air-puff stimulation were significantly prolonged when compared to values without air-puff stimulation, indicating air-puff stimulation-induced antinociception in the spinal cord level. Because this phenomenon was not observed after the LC/SC was bilaterally lesioned, it appears that air-puff stimulation activates the descending pathway from the LC/SC so that nociceptive signals are inhibited in the spinal dorsal horn.

**Figure 10:**
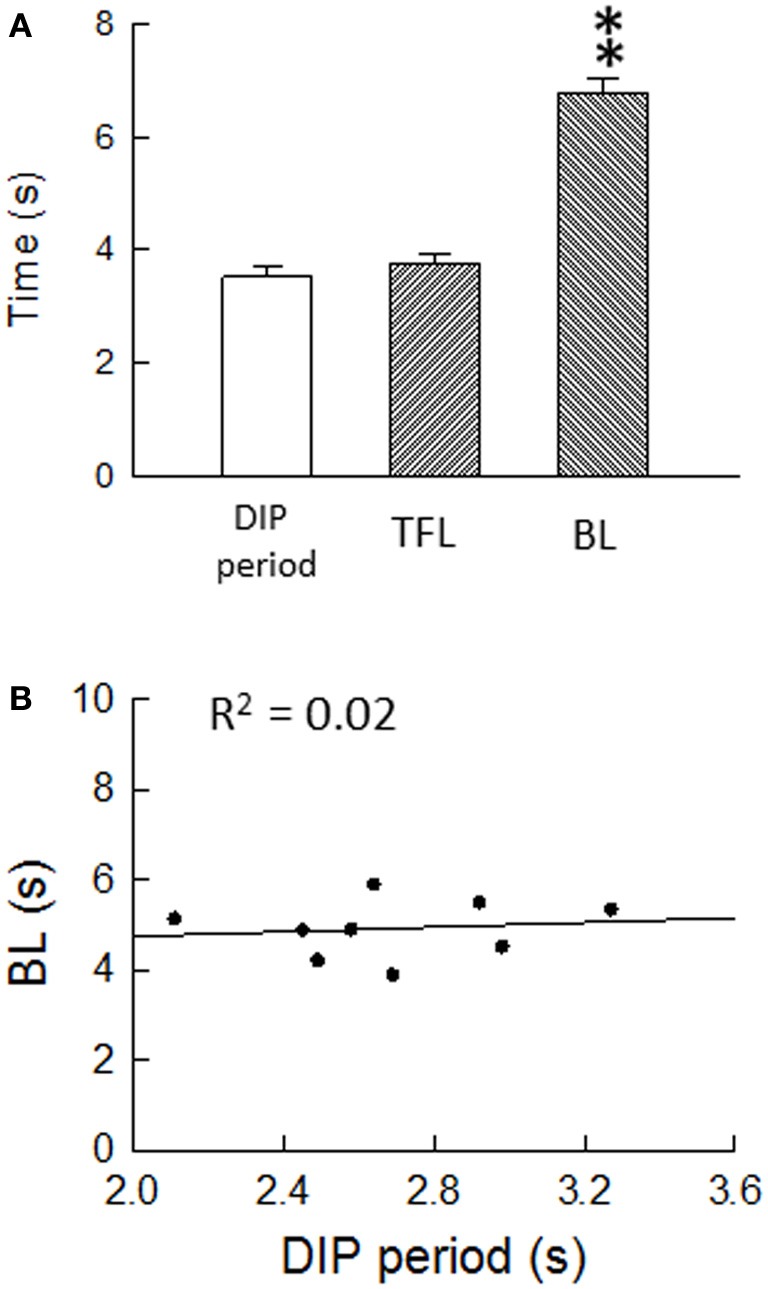
**(A)** Air-puff stimulation-induced the DIP period (*n* = 46), the tail flick latency (*n* = 26) and the bite latency (*n* = 40). ^**^P < 0.01, significantly different from DIP periods and tail flick latencies. Note that there was no significant difference between the DIP period and the tail flick latency, suggesting that the descending and ascending LC/SC systems simultaneously cease activity. **(B)** Regression analysis of the DIP period and the bite latency. Nine points were obtained from nine untreated rats. The regression line corresponds to *y* = 4.24 + 0.26*x*. Note that the bite latency were nearly constant regardless of the change in the DIP period, suggesting that the bite latency are not influenced by the DIP period (Tsuruoka et al., 2011).

Air-puff stimulation induces the DIP, which is a defensive movement, following the startle reaction. It is known that the DIP is mediated by cortical areas, as well as by the LC/SC, in the brain (Cooke and Graziano, 2003). From this finding, it is obvious that the ascending pathway from the LC/SC is activated by air-puff stimulation for inducing the DIP. Because the DIP and the antinociception in the spinal dorsal horn are simultaneous events, it seems that the descending and ascending LC/SC pathways are simultaneously active following air-puff stimulation. Moreover, as shown in Figure 10A, there was no significant difference between the DIP period and the tail flick latency. This result suggests that the activity of the descending and ascending LC/SC systems stop simultaneously.

In addition, as mentioned in the section “Activation of the CSIP by Peripheral Inflammation,” the CSIP is active during an abnormal pain state in the peripheral tissues, such as peripheral inflammation (Tsuruoka and Willis, 1996a,b). This finding indicates that the LC/SC descending mechanism is included in the way of a spino-pontine-spino pathway. In contrast, air-puff stimulation-induced descending inhibition of nociceptive signals in the spinal dorsal horn suggests a top–down modulation of nociceptive signals from the LC/SC to the spinal dorsal horn.

### Air-puff stimulation-induced suppression of bite behavior (Tsuruoka et al., 2011)

Noxious stimuli also induce nociceptive behavior mediated by a higher center of the brain (e.g., Woolfe and MacDonald, 1944). If air-puff stimulation-induced activation of the CSIP inhibits nociceptive signals in the spinal dorsal horn, it will certainly result in suppression of nociceptive behavior. Following heating of the tail, rats whisked their tail and then bit the heat source when tail heating was continued because the tail could not escape from heating by tail flick. In our study, the bite behavior induced by tail heating was a candidate for nociceptive behavior mediated by a higher center of the brain. The bite latency was defined as the time between the onset of heat stimulation and the first motion of bite behavior. Because it was shown that bite behavior was undoubtedly nociceptive and that the bite latency reflected the nociceptive threshold for evoking bite behavior (Tsuruoka et al., 2011), the bite latency could be considered as an indicator for estimating nociception.

Bite latencies with air-puff stimulation were significantly prolonged when compared to values without air-puff stimulation (Tsuruoka et al., 2011). This result indicates air-puff stimulation-induced nociceptive inhibition. Fanselow and Helmstetter (1988) have shown that rats react with a defense response of freezing and a reduction in sensitivity to painful stimulation when they are placed in a situation that has come to be associated with footshock through the process of Pavlovian conditioning. Our study is not the same as that of Fanselow et al. in content; the experimental conditions in our study were different in the following two ways: (1) air-puff stimulation is not painful stimulation and (2) our study did not utilize of Pavlovian conditioning. Indeed, influences of learning the experimental conditions could be excluded in our experiment (Tsuruoka et al., 2011). The air-puff stimulation-induced nociceptive inhibition may be different from nociceptive inhibition under a fear-like condition with respect to the underlying mechanisms and the biological meaning.

Regarding air-puff stimulation-induced nociceptive inhibition, there is a possibility that the air-puff stimulation-induced prolongation of the bite latency is due to decreased interactions in motor response/behavioral output systems, but not in decreased sensory processing. It seems that air-puff stimulation suppresses motor response/behavioral output systems so that the DIP is induced. However, as seen in Figure 10B, the bite latency was held nearly constant regardless of changes in the DIP period, suggesting that the bite latency is not influenced by the DIP period. With the result of air-puff stimulation-induced coeruleospinal antinociception, this finding generates the notion that nociceptive inhibition is an independent component of the air-puff startle response, although the DIP and the nociceptive inhibition are simultaneous behavioral responses in the air-puff startle. We speculate that two air-puff stimulation-induced events, which are suppression in motor response/behavioral output systems and nociceptive inhibition, are a parallel phenomenon, but are not related via cause and effect.

### Involvement of the CSIP in air-puff stimulation-induced suppression of bite behavior (Tsuruoka et al., 2011)

**Figure 11:**
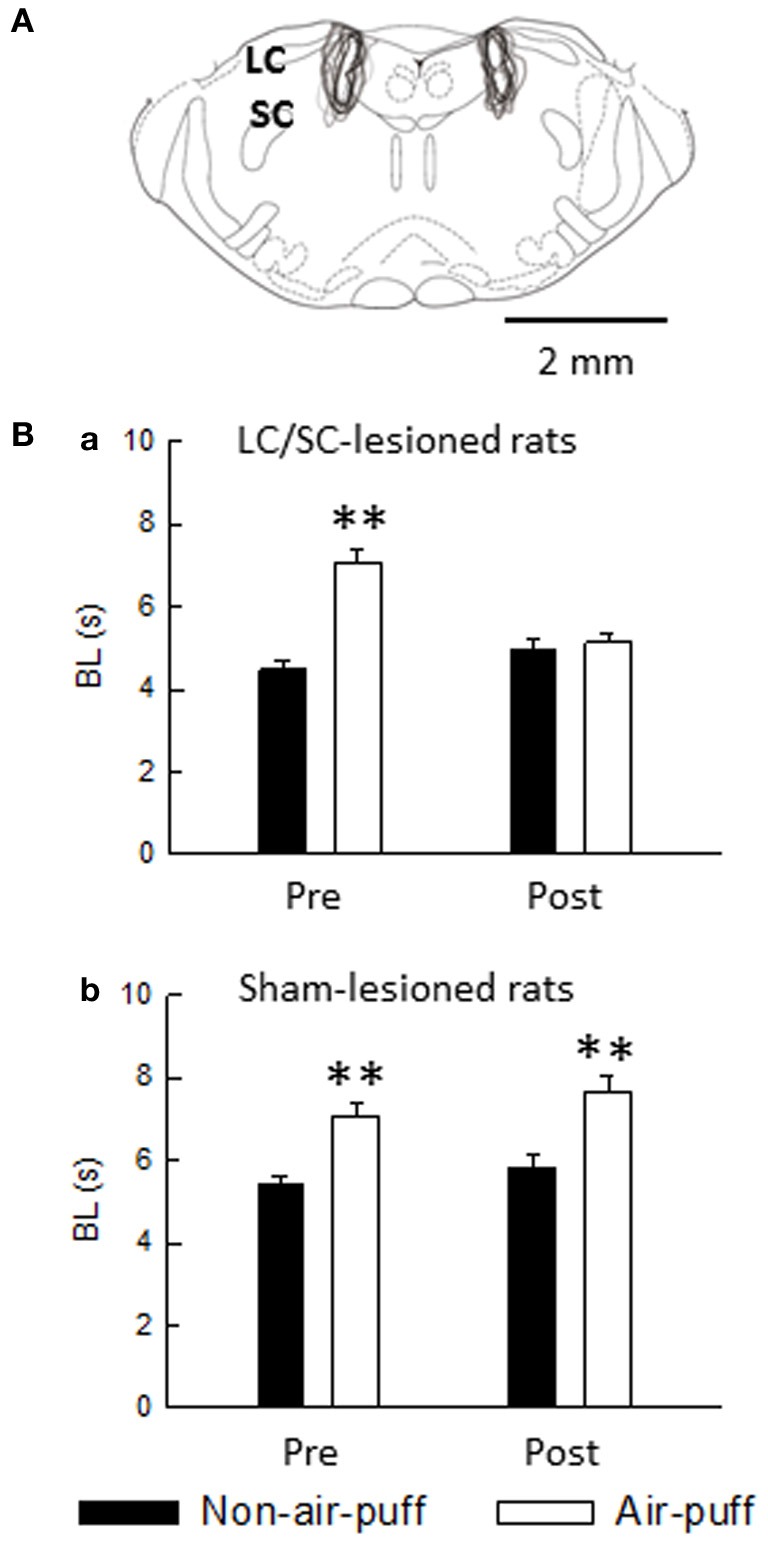
**(A)** Extent of neurotoxin-induced bilateral lesions of the LC/SC (*n* = 10). The rostrocaudal extension was between 0.8 and 1.5 mm, and the LC/SC was always completely destroyed ventrodorsally throughout its rostrocaudal extension. The drawing is simplified from Paxinos and Watson (1998). **(B)** The effect of either bilateral lesions of the LC/SC (**a**, *n* = 10) or sham lesions of the LC/SC (**b**, *n* = 10) on the bite latency. Pre, pre-lesions; Post, post-lesions. ^**^P < 0.01, significantly different from bite latencies of the non-air-puff condition. Note that a significant air-puff stimulation-induced prolongation of the bite latency was not observed in post-lesions of the LC/SC-lesioned rats, whereas bite latencies were significantly prolonged by air-puff stimulation in pre-lesions, suggesting that the LC/SC is involved in air-puff stimulation-induced nociceptive modulation (Tsuruoka et al., 2011).

In the LC/SC-lesioned rats, air-puff stimulation-induced prolongation of bite latencies was not observed in post-lesions, whereas air-puff stimulation significantly prolonged bite latencies in pre-lesions (Figure 11Ba). In contrast, in the sham-lesioned rats, air-puff stimulation significantly prolonged bite latencies in both pre- and post-lesions (Figure 11Bb).

It has been reported that a connection from the dorsomedial hypothalamus through the rostral ventromedial medulla takes part in the circuitry of air-puff stress (Zaretsky et al., 2003) and that the dorsomedial hypothalamus recruits nociceptive-modulating neurons in the rostral ventromedial medulla (de Menezes et al., 2008). We have shown that the LC/SC is involved in the circuitry of air-puff stress (Tsuruoka et al., 2010a). As suggested from the result shown in Figure 11, the LC/SC is also a brain structure involved in the air-puff stimulation-induced nociceptive inhibition mechanism.

**Figure 12:**
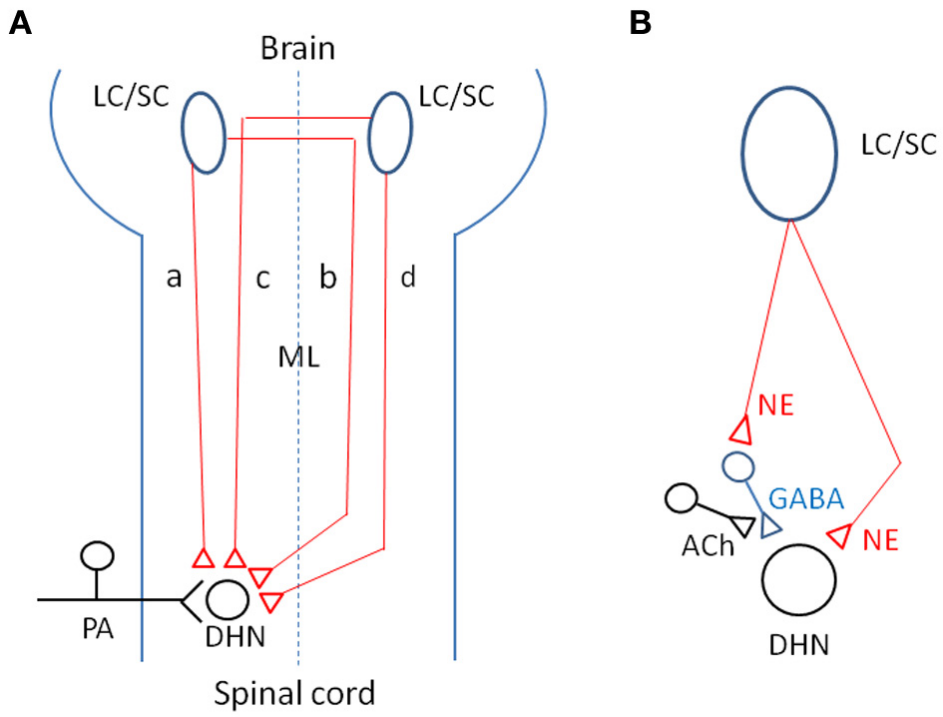
**(A)** Schematic representation of four CSIPs demonstrated. **(a)** In ipsilaterally projecting neurons, axons descend the ipsilateral dorsolateral funiculus or ventrolateral funiculus to terminate in the dorsal horn on the side of the descending projection. **(b)** In ipsilaterally projecting neurons, axons cross the midline within the brain, travel through the contralateral ventrolateral funiculus and recross the midline at spinal segmental levels. **(c)** In contralaterally projecting neurons, axons cross the midline within the brain and travel through the dorsolateral funiculus to terminate in the dorsal horn on the side of the descending projection. **(d)** In contralaterally projecting neurons, axons descend through the ipsilateral ventrolateral funiculus and cross the midline at spinal segmental levels. **(B)** Possible neurotransmitters related to coeruleospinal inhibition of nociceptive signals in the dorsal horn (see text in detail). Keys for the brain and spinal cord are indicated by abbreviations: LC/SC, locus coeruleus/subcoeruleus; ML, midline; PA, primary afferents; DHN, dorsal horn neuron; NE, norepinephrine; ACh, acetylcholine; GABA, gamma-aminobutyric acid.

At this time, as shown in Figure 12A, the following four CSIPs are demonstrated: (1) in ipsilaterally projecting neurons, axons descend the ipsilateral dorsolateral funiculus or ventrolateral funiculus to terminate in the dorsal horn on the side of the descending projection (Sluka and Westlund, 1992); (2) in ipsilaterally projecting neurons, axons cross the midline within the brain, travel through the contralateral ventrolateral funiculus and recross the midline at spinal segmental levels (Jones and Gebhart, 1987); (3) in contralaterally projecting neurons, axons cross the midline within the brain and travel through the dorsolateral funiculus to terminate in the dorsal horn on the side of the descending projection (Clark and Proudfit, 1992); and (4) in contralaterally projecting neurons, axons descend through the ipsilateral ventrolateral funiculus and cross the midline at spinal segmental levels (Tsuruoka et al., 2004). Neurotransmitters related to coeruleospinal inhibition of nociceptive signals are shown in Figure 12B. Norepinephrine released from descending LC/SC neurons is received by α2-adrenoceptor, and nociceptive signals are inhibited pre- or post-synaptically (Willis and Coggeshall, 2004). Inhibitory effects of GABAergic inteneurons are facilitated by cholinergic interneurons (Baba et al., 1998).

### Biological implications of air-puff stimulation-induced nociceptive inhibition

Our results may support the finding reported by Bushnell et al. (2004) that pain sensation is often reduced under an attentional state or a vigilance condition. LC/SC neurons have been implicated in the regulation of attentional states and vigilance (Foote et al., 1983; Aston-Jones et al., 1991). There is evidence that deregulation of the LC-noradrenergic system causes clinical problem in human, such as attention deficit hyperactivity disorder (see Berridge and Waterhouse, 2003). Because induction of the DIP following an air-puff startle reaction is mediated by the LC/SC (Tsuruoka et al., 2010a), the DIP period seems to be an attentional state and a vigilance condition. This notion may be supported by the finding reported by Knapp and Pohorecky (1995) that an air-puff stimulus elicits ultrasonic vocalizations (e.g., 22 kHz), which are thought to reflect an aversive behavioral state following the startle reaction in rats. It is obvious that the air-puff startle response occurs in conscious animals but not in anesthetized animals. Considering these findings, it may be possible to infer that nociceptive inhibition produced by air-puff stimulation forms part of the emotional reaction in animals. During the DIP period, rats probably focus on the judgment of circumstances so that nociceptive signals may be inhibited to extract other sensory information which is essential to the circumstantial judgment. It is likely that sensory information related to the survival of an individual has priority over pain signals. Concerning the function of the LC/SC in the regulation of attentional states and vigilance, we speculate that following the startle reaction the LC/SC suppresses both motor and pain systems for judging circumstances.

### Conflict of interest statement

The authors declare that the research was conducted in the absence of any commercial or financial relationships that could be construed as a potential conflict of interest.
